# How Can Science and Research Work Well? Toward a Critique of *New Public Management Practices* in Academia From a Socio-Philosophical Perspective

**DOI:** 10.3389/frma.2022.791114

**Published:** 2022-04-04

**Authors:** Jan-Philipp Kruse

**Affiliations:** Collaborative Research Centre 1285 “Invectivity. Constellations and Dynamics of disparagement”, Technical University Dresden, Dresden, Germany

**Keywords:** New Public Management, science organization, evaluation, academic precarity, critical theory, social ontology

## Abstract

While *New Public Management* practices (NPM) have been adopted in academia and higher education over the past two decades, this paper is investigating their role in a specifically socio-philosophical way: The preeminent question is what organization of science is likely to make science and research work well in the context of a complex society. The starting point is an obvious intuition: that academia would be “economized” by NPM (basically, that something is coming from the outside and is disturbing the inside). Habermas provides a sophisticated theorization for this intuition. In contrast, the thesis advanced here is that we should consider NPM potentially problematic—but not for descending from economics or administration outside academia. It is because NPM often cannot help research and science to function well. In this (rather “essayistic” than strictly deductive) consideration, I will therefore tentatively discuss an alternative approach that takes up critical intuitions while transposing them into a different setting. If we understand science and research as a *form of life*, a different picture emerges that can still bring immanent standards to bear, but at the same time compose them more broadly. This outlines a socio-philosophical critique of NPM. Accordingly, the decisive factor is not NPM's provenance. What is decisive is that it addresses some organizational problems while at the same time creating new ones. At the end, an outlook is sketched on how the specific situation of NPM allows some hypotheses on academy's [by “academy”, I am referring to the whole research community (like “academia”)] future organization. Ex negativo, it seems likely that qualitative evaluation criteria and creative freedom will have to play a greater role.

## Introduction

*New Public Management* practices (NPM) have been adopted in academia and higher education over the past two decades. “A number of commentators have born witness to the growth of manageriaIization in the public sector” in general, while there are “case stud[ies] of the NPM” in “higher education” as well (Chandler et al., 2002, p. 1052f.). I will approach the question of whether this is more of a *boon or a curse* in a specifically socio-philosophical way. In doing so, I will concentrate on an aspect that lies outside the focus of ethics or philosophy of science and thus complements such studies. The preeminent question is not whether NPM is (ethically) good or bad; it is what organization of science is likely to make science and research work well in the context of a complex society.

In this sense, I would like to understand NPM here as a form of organization. It is a form bearing features that are familiar from business life: “These ideas where strongly influenced by management practices in the private sector” (Kraan et al., 2010, p. 73), founded “on themes of disaggregation, competition, and incentivization” (Dunleavy et al., 2005, p. 467). For example, the allocation of resources tends to be regulated on the basis of criteria that are relatively easy to measure in quantitative terms (“a concern for more explicit and measurable standards of performance”, Chandler et al., 2002, p. 1054). In an academic context, this could be related to how many articles have been published, how many theses have been supervised, and so on.

The inquiry starts with an argument by Habermas and initially follows the critical perspective on NPM that derives from it. From this view, the problem is that NPM's criteria have not evolved “organically” from academic discourse. I draw on this passage because it fits well with an obvious intuition: that academia would be “economized” by NPM. Habermas provides a sophisticated theorization for this intuition, while the paper will not be primarily about Habermas.

The economization intuition serves more as a contrast in the second step, for it rests on problematic presuppositions. With their problematization, the view on NPM also changes. The thesis advanced here is that we should consider them potentially problematic—but not because they descend from economics or administration outside academia. It is because NPM often cannot help research and science to function well. With this critique of NPM, I will follow those studies that already consider NPM as a paradigm of the past:

“[I]n the 1990s and early 21st century, ‘New Public Management' was the dominant theme. Today, public administration is moving in new directions.” These new directions “are focusing on the quality of services for citizens … and on the efficiency of administration”.^1^

The “character of the post-NPM regime is currently being formed”: “New developments accrete and accumulate while older trends are still playing out and apparently flourishing.” Also, “NPM ideas are still gaining influence in previously rather resistant countries, such as Japan”, as Dunleavy et al. have already stated in 2005 (Dunleavy et al., 2005, p. 467f.). Likewise, they have already hinted to changing preferences in social organization, including a “needs-based holism” (op. cit., 480).

The socio-philosophical point is that this development takes place for good, understandable reasons: because it is precisely the dimension of “needs”, or in general the dimension of *quality* that often cannot be satisfied by NPM, also on the academic terrain.

I arrive at a readjusted picture by discussing Habermas' presuppositions with a normative-functionalist twist. With this twist, criticizing or assessing NPM is less about crossing boundaries in the Habermasian sense: about breaking through cultural (here: academic) traditions and thus unsettling them.^2^ Rather, the question is which planning activities can help science and research to function well (and which ones cannot). The actualized perspective intends to make clearer what might be problematic about NPM, and why.

In more detail, it is the connection between planning activity and education as conceived by Habermas that serves as an introduction:

“educational planning … produces a universal pressure for legitimation in a sphere that was once distinguished precisely for its power of self-legitimation” (Habermas, (1988) [1976], p. 71).

Since I am concerned with a systematic clarification, I will not go too deeply into Habermas debates. Rather, I will elaborate on the cited passage that concisely understands education as a “nature-like” (ibid.) tradition and therefore must view NPM as something coming from the outside, which can potentially destabilize the inner logic of its development.

In Section *NPM From a Socio-Philosophical Approach*, I will briefly reconstruct the notion of such an “intrinsic logic” and relate this notion to science and research. In doing so, I roughly follow the intuition that something is coming from the outside and disturbing the inside. The question then is whether a certain form of organization can be explained immanently from the developmental logics of research and science or not. Where this fails, it must appear as a disturbing factor rather than as an organizational aid. In a next step, I will show that the notion obtained, however, depends on very specific premises. It arises from (heuristically partially helpful, yet) problematic distinctions in the field of social ontology: concepts such as “intrinsic logic” or “system” first distinguish social spheres in order to then ask about their relation to each other.

In Section *Socio-Philosophical Critique of Academic NPM*, I will therefore tentatively discuss an alternative approach that takes up critical intuitions of the “intrinsic logic” model while transposing them into a different setting. If we understand science and research as a *form of life* (cf. Jaeggi, 2018), a different picture emerges that can still bring immanent standards to bear, but at the same time compose them more broadly. With it, we can think of science and research as spheres deeply integrated into society with simultaneously immanent standards. The challenge then is to elaborate these standards in a self-reflexive societal discourse and to defend them against “overforming”. By overforming, I mean regulations that (regardless of their origin) do not help science and research to function well.

At the end, an outlook is sketched on how the specific situation of NPM—between solving old problems and creating new ones—allows some hypotheses on academy's future organization. Ex negativo, it seems likely that qualitative evaluation criteria and creative freedom will have to play a greater role.^3^

## NPM From a Socio-Philosophical Approach

### What Is Academic NPM?

I would like to start with a remark about the interaction of definition and critique. Only when we adequately conceptualize NPM can we understand exactly what is problematic about them. The focus of the main section is therefore on conceptual elements of NPM—if these are not problematic because they come from outside the academy, why are they problematic?

In this respect, it does not seem conducive to anticipate a strong definition of NPM here. That said, of course we should know what we are talking about from the outset. A 2010 OECD report succinctly summarizes five characteristics of an NPM “solid core”:

“Separation of execution from policy development ... More autonomy for [...] managers in operational management ... Steering and control of executive agencies on the basis of measured output ... Budgeting on the basis of measured output [and] ... Outsourcing of intermediate production to the market” (Kraan et al., 2010, p. 55).^4^

From an academic perspective, several aspects of this are questionable. Experience with NPM has taught that it has created certain selection mechanisms whose relation to scientific excellence is not clear. Enders and Westerheijden even claim that there would be an “absence of political will to establish robust evidence” (with emphasis “on quality in teaching and learning”, Enders and Westerheijden, 2014, p. 190).

On the one hand, one could argue that some scientific achievements are not well quantifiable. For example, in basic research, a lot of time has to be invested without automatically resulting in more findings. This type of research is then at a disadvantage, although from a scientific perspective it is not clear why it should be worth less than more measurable projects.

On the other hand—and this is important for a critical perspective—, it cannot be argued that NPM has made things worse everywhere in the academy that were clearly better before. NPM's benchmark element has also brought with it an element of “promise”. For example, there is a known tendency for theories to insulate themselves from discourse, or for camps to form. Hence the (bad) joke that a theory is not disproven until its last proponent has died. In this regard, NPMs have promised more dynamism: researchers must apply for funding. Such an application has to be justified to the community. Therefore, it is harder to pursue idiosyncratic ideas.^5^ Of course, the objections already mentioned can be raised here as well. A research project that sells badly does not necessarily have to be a bad research project.

So, the situation is definitely diffuse. What seems clear is that there is a problem with NPM; but also, that academy had not been perfectly organized before NPM either. Against this background, how can we consistently criticize NPM? I will argue below that the socio-philosophical tradition offers some interesting clues for this. However, for our purposes relevant here, it takes a wrong turn when it focuses on proprietary developmental logics. For a critique of NPM, I argue, it makes more sense to follow a normative-functionalist twist.

### Looking at NPM With Habermas

My question is both systematic and socio-philosophical. The initial focus on an argument by Habermas in the context of his theory of legitimation^6^ has to do with the fact that this argument seems to be particularly interesting when it comes to NPM. Philologically, I will classify it only as far as it seems necessary for the clarification of New Public Management practices.

Habermas has commented in various ways on the structure of knowledge and institutions of knowledge. Already his inaugural lecture from 1965 distinguishes different “Erkenntnisinteressen” (epistemic interests). In *Cognition and Interest* (Habermas, (1987) [1972]), psychoanalysis is elaborated as a model of self-reflexive cognition.^7^

The idea of a “nature-like” development of academy is considered there in a certain way: however, more as a self-understanding than as a logic of development. With Husserl, Habermas exemplifies this understanding of what it would truly mean to work scientifically: the notion of a “pure” theory. That notion is then criticized—theory is not without interest (and where it claims to be, it deceives itself). In Habermas' engagement with epistemic interests, Husserl's position therefore marks an intermediate step toward the self-reflexive model that psychoanalysis would exemplify;^8^ while in his later works, the perspective has turned more toward developmental logics. Social subsystems differentiate and cluster around a realm of cultural understandings and traditions—the so-called “lifeworld.” The danger of “colonization” of this lifeworld by system imperatives is in the center of attention, because it threatens resources of meaning that could not simply be reproduced.^9^ In the light of Habermas' late philosophy of religion, it is even suggested that he considers the loss of such meaning resources irreversible (for if it were not, there would be no urgent motive to deploy strong semantics from religion for civil discourse, as Habermas discusses there).^10^

In any case, a bridge can be built from here. The view that cultural resources of meaning are a special type of resource already plays an important role in the theory of the legitimation crisis:

“Contra contemporary views that state manipulation is potentially unlimited, Habermas insists on the willfulness (*Eigensinn*) of the cultural system in imposing limits on administrative power” (Nullmeier, 2017, p. 315).

The cultural sphere, he argues, is somehow stubborn—i.e., what makes sense to it, and what does not, cannot be prescribed—and therefore could not be manipulated by ideology unlimitedly (Limitation Thesis).

Interestingly, Habermas cites education as an example in this context. As a sphere essentially organized out of itself, it would be damaged in a specific way if it became the object of administrative planning imposed from outside. That damage cannot be “direct” for Habermas in view of the above-mentioned limitation thesis (that culture sets a limit for administrative manipulation), as if education would be directly broken by external administration. The point is that a pressure of legitimacy is created:

“Whereas school administrations formerly merely had to codify a canon that had taken shape in an unplanned, nature-like manner, present curriculum planning ... produces a universal pressure for legitimation in a sphere that was once distinguished precisely for its power of self-legitimation” (Habermas, (1988) [1976], p. 71).

Even if, as announced, I do not want to go too deeply into Habermas-internal debates, it should at least be mentioned here that with this, the complexity of Habermas' theory of social rationalization is already indicated.^11^ On the one hand, it is about colonization, in the sense of crossing a boundary: the “grammar” of the one overwrites that of the other sphere of life. In this logic, one can say that spheres of life are “economized”.

On the other hand, the tendency of a “strange self-devaluation of the lifeworld” (Celikates and Pollmann, 2006, p. 109 [transl. jpk]) is described. This tendency is shown by the term “universal” in the passage quoted above. The first part of the quotation reads in full:

“Whereas school administrations formerly merely had to codify a canon that had taken shape in an unplanned, nature-like manner, present curriculum planning is based on the premise that traditional patterns could as well be otherwise” (Habermas, (1988) [1976], p. 71).

For our purpose, I would like to summarize it like this: social modernization puts cultural traditions under pressure anyway; they have to justify themselves simply because alternative approaches to similar problems become known or at least conceivable. As a result, they are less able to counter colonializing tendencies: an area that has lost its own legitimacy can be reshaped all the more easily. In the context of colonization, “reshaped” means that the logic of another social sphere is to be imposed.

### Intrinsic Logics and Evolutionary Logics

In the following, I will concentrate on the thesis of an intrinsic logic and reformulate it independently of Habermas in such a way that it contributes to the clarification of our question—about NPM whether being *boon or curse*.

Habermas talks about culture as a sphere on the one hand, while citing curricula as examples on the other.^12^ Our concern lies in between: neither is it about culture at all, nor about school education in the narrow sense. It is about academia or “academy”, that is, about research and university teaching. What is taught and what is researched there is not subject to direct planning or control in western societies. On the one hand, a certain canon has developed, which on the other hand is updated by current research. It is shaped by rather soft control instances like professional publics, co-workers and students. Editors and publishers accept or reject articles. The faculty decides who is promoted into its ranks and who is not. Students will ask themselves whether a course has helped them understand its subject.

With these examples, I would like to point to the contours of something like a logic of its own, an intrinsic logic: academy knows itself how to organize certain things in a reasonable way. “Intrinsic” can mean at least two things. On the one hand, it can be understood in evolutionary terms. We will first follow this interpretation in order to arrive at a normative-functionalist view.

The idea of an intrinsic logic seems worth examining, if not promising, in order to gain resources for a substantive critique of NPM. My concern at this point is where exactly the focus of further investigation should lie. One obvious option would be to focus on the development of such an intrinsic logic. For Habermas, the concern with developmental logics is clearly related to the fact that he has increasingly distinguished between spheres of society.^13^ The analytical distinction is understood as tracing a process of social differentiation. Yet, with this comes a fundamental methodological difficulty; and focusing on development generates comparatively little thrust for a critique of NPM.

#### Methodological Vagueness

I would only like to touch on the methodological difficulty here. It consists in the fact that the distinction between different areas of society can be analytically productive on the one hand, but on the other hand (like every abstraction) it also comes with a price. Concentrating on the proprietary developmental path of a societal sub-area (which thereby gains shape as a sub-area) is also concentrating on what is specific. The economy is then something different from law, which is, of course, plausible—here money or profit plays the first role, there it is the code of laws. However, both areas are at the same time parts of social life in toto. Economics and law could not function if they did not also take into account human needs that are not economic or legal in the narrow sense. A classic example of this is the logic of contract. It makes little sense to have a contract about contract compliance. That is, the contract regulates a particular matter in the medium of law. However, the expectation that agreements will normally be kept cannot be legally enforced. A certain good will has to be presupposed to some degree, but it is not sensu stricto something legal or economic. The focus on developing a logic of one's own exacerbates this methodological difficulty. After all, good will has not developed specifically, in the sense of the differentiation of a certain mentality for a certain social subsystem. It is conditional, but not condition-specific, which means that good will is needed yet not (exclusively) constituted in subsystems.^14^

This methodological vagueness strikes when it comes to NPM and academy, although the idea of an intrinsic logic seems initially promising for criticizing NPM. We have not yet defined NPM in detail, but we have already hinted at a direction: it is about forms of organization that have made a career in another area of society (primarily the economy) and are now expected to succeed in academia as well. Prima facie, it seems plausible to invoke some sort of proprietary logic against this. Research and teaching are different from business management or public administration. However, if a proprietary logic is conceived as a proprietary development path, two difficulties arise for the argumentation.

First, the contract example has pointed out that the conditions for success of a practice do not have to originate in that practice itself—they too can come “from outside” in some way, like NPM. Consequently, if academy does not function well, the crux of the matter is not that there is an “invasion,”^15^ that something comes from outside and disrupts a “nature-like” development.

Of course, such an invasion or colonization has a certain explanatory value. It can be (and is not improbable) that certain “invasions” actually lead to severe problems. Just one example: all over the world, the precarious working conditions of young scientists are criticized. This precariousness is not part of academy's own tradition. It is true that researchers do not generally strive for wealth; but no one would say that poverty is an ingrediency of good science. Rather, it appears that considerations from (neoliberal) economic psychology have been transferred to the sphere of science: existential pressure as a motivational factor. From research interviews with academics and administrators, Chandler et al. cite the following narrative:

“An excessive feeling of pressure and demand … that distorts your pattern of living, thinking and feelings about yourself and you are not relaxed—you no longer live in the present; you are no longer living life for the self … There is not enough time for yourself to recreate yourself” (Chandler et al., 2002, p. 1058).

It is obvious that such developments lead to human suffering: the “human cost wrought by the introduction of the New Public Management” (op. cit., 1051). However, at least from the internal perspective of the sciences, it is also quite obvious that they hinder rather than promote scientific excellence. Someone who has to seriously worry about how to pay the next rent is simply distracted. Anxiety here is not a stimulant, but a proven obstructive factor for intelligence and creativity. An NPM that seeks to establish and expand precarity as a condition of work is just not helpful at this point—and it comes “from the outside”.

Nevertheless, this view falls short for our purpose. For how would one defend an alternative to the NPM outlined? Good working conditions can be specified for science, but at the same time they are not specific to science. A good working climate will be different in detail than, for example, in the insurance industry. At the same time, there are great similarities that indicate that much of what is relevant here is transverse to social differentiation. Successful interactions, trust, teamwork, these things are grounded in socialization. Critical thrust, however, cannot be gained by playing childhood off against professional life (as if childhood were being colonized). Both with the playdate in kindergarten and with the invitation to the scientific lecture, I wish that I am treated with respect as a guest. The punch line is: if I am treated disrespectfully, I don't get irritated because it violates a contingent rule of behavior that I grew to cherish as a child. The other way around: I learned respect as a child because this norm can structure situations in a meaningful way. In the practice of science, therefore, it acquires specific relevance: for example, people let each other finish and listen to each other. My point is that a norm like respect serves a function for academy that is specified but not proprietarily developed. That's why talking about developmental logics is not the most appropriate when criticizing NPM. It may well be that NPMs violate academic tradition. But there are many conceivable cases in which the good of the violated tradition is not to be a peculiar tradition. Rather, their good is that they have helped academy function well.

#### Little Thrust

This brings us to a second aspect. The developmental logic approach is not only not clear-cut when it comes to the provenance of regulating normative elements like respect. From the other side, the notion of a differentiated intrinsic logic—even if it were consistent—is not very appealing for our case. We are familiar with caveats of this type from the economic debate: what is “good” for the economy need not be good for people. The economy is about maximizing profit. But maximum profit does not mean maximum quality of life. As the ecological crisis shows, the two can even diverge quite drastically.

Similar reservations have long existed in academy. Is it a good thing at all for science to manage itself and develop independently? The history of the 20th century, at the latest, has sown doubts about this.

Two points: First, I would like to refer just briefly to the example of basic research in totalitarian regimes. Where were researchers forced to do something, and where were they themselves a driving force, for example in experiments on humans? The answer to this question is not always easy. On the other hand, as natural sciences are advancing, a new kind of skepticism about science has emerged. This skepticism about elaborate experiments is not completely absurd. I am not talking about conspiracy theories here; I am talking about concerns raised even by Nobel laureates.^16^ At the heart of the matter is the fact that scientific experiments now have the potential to alter or even destroy the whole planet (and thus the very basis of human life). A nuclear bomb can irradiate an atoll. The particle accelerator CERN could, in the most unfortunate case, create a black hole that swallows Earth. This case is considered extremely unlikely, yet its probability is >0 or baseline. It is extremely unlikely, though not impossible. Nevertheless, this literally global risk has never been brought to a vote. To take it was a decision made by the researches involved. From the logic of physics discourse, it seemed reasonable to build an even more powerful particle accelerator. CERN very likely poses an acceptable risk. My point is that it indicates a threshold. A “l'art pour l'art” of academy^17^ is (finally) implausible against its background, because its activities have an impact on the foundations of society (the scientific manifestation of the Anthropocene).

Finally, certain things can be described as academic culture that don't require much discussion: from medieval hats and titles to fraternities, there are phenomena that are obviously controversial. My concern at this point is only with the controversy itself—that there is one should be uncontroversial. Also, university life before NPM was not necessarily “good”; in Germany, the problems were almost opposite to the situation today. Nowadays, students complain about academy becoming more and more like school. Back then, there was sometimes suffering from too little structure.

In summary, the idea of an intrinsic logic does seem attractive for a critique of NPM. Academy has learned how it should organize itself. Outside influence would then be disturbing. However, the idea of intrinsic logic, understood as a proprietary development path, faces at least two difficulties. It is not sharp, and it is not itself unproblematic in normative terms.

In what follows, I will argue for a normative-functionalist twist. With it, we will try to circumvent the methodological difficulties mentioned above. At the same time, it will strive to outline a valid argumentative basis for the critique of NPM.

### When Academic NPM Does Not Function Well

Jaeggi's (2018) *Critique of Forms of Life* (CFL) comes with a number of striking examples. One of these is the practice of medicine. A doctor who did not strive to heal her patients would (first) not only fail to be a good doctor. We would (secondly) not even understand what that person is actually doing in a medical context.^18^

On this basis, then, we could criticize a doctor who calls herself a doctor but does not strive to heal. At first glance, it may seem that we are moving here quite close to the sketch obtained with Habermas. In fact, Jaeggi connects to him in a variety of ways. She, too, is concerned with something like intrinsic logics, namely with the constitution of forms of life. It is trivial that a pilot is unlikely to be a good doctor. Jaeggi's punch line is: the person who calls herself a doctor, but then fails to live up to the standards of medicine is a bad doctor.

She also adds a Hegelian element to this Aristotelian one, which is the development of forms of life in the course of time. So, in a sense, one could say that Jaeggi's *Critique of Forms of Life* is also interested in logics of development.

The difference relevant for our purpose is that the concept of life form is transverse to strong assumptions concerning social differentiation, and the social ontology associated with it. Small things are a form of life (like family). Big things can be characterized as form of life (like capitalism). One can imagine how life forms overlap. The concept is largely scalable.

Against this background, Jaeggi looks at the specific functioning of certain areas of life, which have their own history as formed spheres of life; at the same time, the good or bad functioning of a life form is not as narrowly defined as in approaches that differentiate social systems. The life-form concept, as noted, is freely scalable and consequently allows overlapping. Therefore, good functioning in one form of life cannot be completely abstracted from other spheres of life. A good family in Jaeggi's sense is a family that manages the demands placed on it non-regressively in the context of a society (and not against that society).^19^

Thus, even if the form aspect is rather loosely conceived in Jaeggi's forms of life, it is also not dispensable. For specific or even intrinsic standards follow from the concept of form—precisely the standards of a form of life. So, there is also the notion of an intrinsic logic here. And this logic is said to have developed in the course of historical learning processes (the Hegelian element). For at least two reasons, Jaeggi's approach appears nevertheless more promising for the critique of NPM.

For one thing, as already mentioned, her concept of form is initially less strict than the distinction into social subsystems (i.e., scalable and overlapping). For another, her account is characterized by explaining forms as contradictions. In this respect, the concept of form is defined stronger. A form of life does not get into (external) contradictions, but is contradictorily constituted as a life-form.^20^ Jaeggi thus aims at a more complex conception of the relationship between social norms and practices. In her conception, it is not the case that a norm—quasi binary—is realized or not realized. Rather, she argues, forms of life are characterized by contradictory realizations of norms. To give an example: in the market economy, freedom plays a thoroughly important role. Nevertheless, it does not establish freedom for all participants. And this would not be a coincidence in Jaeggi's eyes, but one aspect is related to the other. The freedom of some has something to do with the lack of freedom of others. In this respect, the norm of freedom would then be realized contradictorily. I will not go deeply into the concept of contradiction here. The point is that forms of life are loosely defined; however, through the concept of contradiction they also acquire an intrinsic nature. With it, the constitution of a form of life is described.

What interests me about Jaeggi's CFL in this context is that it provides criteria for a critique of NPM. These criteria are also oriented toward notions of an intrinsic logic and development. But they are at the same time less threatened by a lack of sharpness and a lack of thrust (see above). We might say somewhat polemically: they are not threatened by either a conservative or a technocratic misunderstanding. Conservative in this sense would be to play off academy's tradition against NPM (problem: not everything about the tradition was good). “Technocratic” means to focus on the logic of social subsystems without sufficiently considering their dependence on and embeddedness in overarching social structures (problem: proprietary development paths are not good merely because they can be described as proprietary).

The readjusted question then, with respect to academy, is: does an academic practice work well? With Jaeggi, we can answer: the criteria of this question are not arbitrary. On the one hand, they derive from the history of academy's development. On the other hand, academy has, after all, done something in this history: it has researched and taught. Its history is thus the background of the pragmatic self-understanding of researchers, which results from their respective research practices. Taken together, we can speak of a horizon of expectations or profile of requirements. This has neither a historical bias, nor an actualist one. The argument “We have always done it this way” would be valid if the learning experience sedimented in academic self-understanding proves to be meaningful for current challenges, and is able to evolve on them.

With this approach, the historical dimension is not dropped, but conceived in a more complex way. Adaptations along normative ideas come to the center of attention. In Jaeggi's case, the individual process stages of the historical dimension are interpreted with the concept of contradiction: a norm N is realized in practice P in a contradictory way.^21^ This could mean, for example, that it is constitutive of practice, but that in the particular way of constitution it is at the same time deficient in realization. This has the merit of thinking together the complexity and confusion of phenomena with the notion of directed social change.

At the same time, however, this results in a methodological burden, especially for our purpose. The concept of contradiction is to be understood here rather reconstructively. It describes, so to speak, entangled practices, which in the end (that is the reconstructive part) move along certain, normatively composed lines of adaptation. Therefore, no simple conclusions can be derived for the individual situation (such as: norm N' is not in place, but it should be in place).

With the help of the perspective that CFL opens up, it is not so much possible to advocate certain norms; rather, the perspective is meant to accompany transformation processes:

“Criticism of forms of life … as I understand it here … is not intended as advocacy of a relapse into premodern paternalism, but instead as an exploration of the conditions of what can be conceived in the tradition of critical theory as a ferment of individual and collective *emancipation processes*” (Jaeggi, 2018, p. xi).

N and P are knotted together, so to speak. Thus, critique cannot be a matter of inaugurating N, but rather of developing the constellation of N and P further. In other words: how can P function better?

With this in mind, in the following section I will discuss some proposals that are meant to serve as a critique of NPM by tentatively suggesting a more advanced perspective.

## Socio-Philosophical Critique of Academic NPM

How can academy function well? By evolving its norms and practices, we can say in general terms, on the basis of the previous chapters. This unfolding can be called social learning; both (normative elements and practice structures) will change in the process. We are then not advocating a particular norm or focusing solely on empirical trends in the development of practices; we are concerned with a change that proves to be, in Jaeggi's words, “non-regressive,” (Jaeggi, 2018, p. 286) that is, further developing the connection between practices and norms.

On the one hand, as I said, it is a matter of developing connections (rather than advocating a particular norm). For another, such connections can be contextualized differently with Jaeggi's approach. While Habermas focuses on interactions of social spheres, Jaeggi suggests keeping the scale fluid instead. The family life-form has evolved in a somewhat proprietary way, displaying eo ipso a certain intrinsic logic. At the same time, contemporary families are families living in a capitalist form of life.^22^ To make this a little more vivid: for a family, after all, one of its concerns is subsistence. It has a leeway as to how exactly it organizes this subsistence. And how the room for maneuver is exercised has something to do with the “learning history” of families (what did work out? What didn't?). At the same time, that leeway is also economically modulated. Who is doing (which) wage labor? Who takes on what share of care work? Most families cannot decide such questions ad libitum, but have to follow economic constraints. In this respect, one could say that it is part of capitalism's form of life to organize family life along economic lines as well. To a certain extent, this perspective can be arrived at with both Habermas and Jaeggi. Though, as already noted, Jaeggi's approach seems to me to take us a bit further here. For Habermas' *Theory of Communicative Action* (Habermas, (1984/1987) [1981]), economics is allowed to be economistic as long as it does not pervasively spill over into other areas of society. Jaeggi's CFL instead asks (monistically in this respect) about *the* good life. In the end, a family could probably not function well (ultimately not be able to learn) in an economistic economy (even if that economy leaves families some leeway, some room for maneuver), as this economy will then not function well itself. Technically speaking, both: conditions of success of life forms and potential pathologies can be discussed with Jaeggi transversely to strict social differentiation.^23^

I would like to let the sketched socio-philosophical perspective take its course here. The threading goes like this: the institute of a free science requires some sort of quality control. At this point, NPM has effects in an important and at the same time interesting way. The picture becomes more differentiated because NPM seems to be highly problematic in this respect, but at the same time it connects to existing problems of academic organization. The fact that it is not particularly successful in solving academic organizational problems (aiding academy to function well), but appears problematic itself, I discuss on the basis of two condensed paragraphs of enumerative character. First, I identify NPM's quantitative manner as problematic, then the “usability” of an academy shaped by NPM. In sum, NPM is found to be *exaggerated* and *one-sided*. The section concludes with a tentative look to the future. From the critical perspective developed, it seems reasonable to give greater weight to both qualitative evaluation criteria and creative freedom.

Above, it has already been quoted that NPM would rather belong to the past. When, in contrast to NPM, there is talk of a tendency toward more “quality of services for citizens ... and on the efficiency of administration,”^24^ this is already an indication of how NPM may not have been sufficient. Let us follow this track with the socio-philosophical setting as it has been unfolded so far.

The academy has a long tradition of self-administration, up to its own jurisdiction. What interests me most here is the organization of research and teaching. On the one hand, research and teaching should be free. In many western countries this is stated in the constitution (in Germany, § 5 GG). On the other hand, this liberty requires a form of indirect quality control. The lower the access to the distribution of knowledge, the more important is some sort of quality control. In the natural sciences, that is often a matter of replicability, whereas in the humanities, argumentative plausibility is in the foreground.

One can say that this is about a scientific ethos, or norm: of course, scientific publications should be correct. But why should they be? So that research and teaching can function well. Students who learn incorrect knowledge are not likely to be good graduates. Research that is based on false assumptions has less chance of being successful. Finally, these aspects also play a role in filling academic positions. Applicants should have promising résumés.

I would like to interpret it as an effect of NPM that quantitative criteria have been anchored in these areas in many places, or at least are emphasized more strongly. In a sense, the introduction of NPM came from outside the academy; it is a bundle of concepts originating in the business world. Nevertheless, it would fall short to speak of an “economization of the academy” and to make it the center of critique of NPM. At least some of the measures that have come with NPM can be charitably reconstructed as addressing academy's existing problems. Academic NPM then means trying to structure the function of self-governance (including normatively) better than it has been in the past. Ideally, for example, a selection or application process could be made more transparent and efficient. It would then be less about intangibles such as a good network and more about demonstrable performance. In a way, NPM comes from the outside, but it addresses immanent organizational problems.

We therefore cannot avoid looking at it in detail. For at least three (overlapping) reasons, academic organization *via* NPM does not always work well, because quantitative criteria do not seem to be appropriate, or only to a limited extent:

- Preconditions: a quantitatively contested competition includes the idea of equal access conditions. Otherwise, the result of the competition would not be a reliable indicator. For a variety of reasons, however, this access is not always equal. For the sake of brevity, I would like to give just one very simple example: access to publication opportunities, which can also be a financial issue (for example, the printing costs of monographs or Open Access fees).
- Measurability: some scientific achievements are easier to measure quantitatively, others are more difficult. Basic research, for example, can be laborious and yield little tangible results. Failures and falsifications may be less attractive to journals, but still relevant to the research process.
- Normalization tendency: taken together, there is a certain normalization tendency. This means that certain project proposals will be more successful without necessarily being scientifically “better”—because they better fit the quantitative selection.

Secondly, the working conditions within the academy can be examined under the heading of “quality of service”. Does NPM bring about good “usability” of academic institutions for researchers? Four points suggest that academic NPM does not always function well in this respect:

- Frictional costs: academic NPM is costly in two respects. It requires time and attention on the part of the evaluators, which could otherwise benefit their own research. On the part of the evaluated, it involves optimizing the research profile and appearance—again, things that are not core to research.
- Ubiquity of testing: due to the advanced implementation of NPM, it is the case that a kind of “test drive” (Ronell, 1993) hardly ever comes to an end. On the one hand, this is of course plausible. After all, where is it not beneficial to test whether a project is promising, whether those involved are qualified, etc.? On the other hand, this also results in little room for creative experimentation.
- Displacement: in general, unconventional researcher biographies come under pressure. It is in the nature of things that career changers have a shorter publication list. But one can also imagine the paradoxical case of a researcher writing particularly demanding papers—advanced and, precisely for that reason, difficult to digest. Such studies are often read less frequently and cited less often. Even Kant and Hegel had a similar experience in the beginning. And in the recent attention economy, such effects are likely to intensify. NPM's selection does not help such researchers, they tend to be sorted out. Then potential for academy is lost.
- Precarization: finally, NPM has a downside for working conditions when it comes to the distribution of funds. Certain projects are selected and awarded (according to debatable criteria), that is one thing. The other is: not selected researchers sometimes have very few resources. In certain disciplines, there are few permanent positions. Economic precarity has become a real threat to many young scientists. From a normative point of view, this precarity is not to be wished on anyone—that is trivial. But precarity is also a hindrance to research. It is well known that existential anxiety inhibits cognition—curiosity, creativity, and even IQ in the last instance (see Mani et al., 2013). And where this anxiety is observed by students, it is precisely the most gifted of a cohort who look around for other occupations. For them, after all, there is no reason to expose themselves to the danger of precariousness.

Taken together, a critical but also differentiated picture of NPM emerges, if we understand it as a form of organization in academia. In a sense, NPM comes to academia from the outside, while it ties in with internal problems. In the process, a variety of new problems arise. For example, NPM's emphasis on quantitative output can in some ways be interpreted meritocratically. A meritocratic ethos fits well with something like the intrinsic logic of science. However, in many places NPM has exaggerated and one-sided this ethos. By “exaggerated,” I mean that there are limits in principle to the evaluability of research approaches. A certain scope for experimentation and trial and error—without knowing in advance exactly what it will all lead to—is also part of the form of life of the sciences, but is not taken into account by NPM. By “one-sided,” I mean NPM's focus on quantifiable properties. Not all components of the scientific process can be equally quantified. There are aspects of research that can be evaluated, but not quantitatively.

With these considerations in mind, we can say that NPM addresses academia's problems and at the same time, in the way it organizes these problems, can itself become a hindrance to research. In such cases, it is not able to help academia function well or better. In the areas of basic research, advanced theory, and junior scholars, for example, there are good reasons to consider NPM problematic.

With this critique, problematic aspects of academic NPM can be described, without addressing all of academia's potential problems. The organizational form of NPM competes with other organizational forms, and often there is overlap. Problems of their own kind can arise from the fact that NPM plays a role, but is then not consistently implemented. For example, mixed selection procedures can lead to frustration among those involved (when optimization attempts are based on quantitative criteria that do not end up being the deciding factor).

For a look into the future, I will nevertheless focus on the role of NPM in recent academia. The assessment that NPM is a paradigm of the past has already been quoted. From the critique of—or the problems with—NPM, one can indirectly infer where the specific challenges of future organizational forms lie.

In view of NPM's quantitative selection, it is obvious to give more weight to qualitative criteria. They, too, are time-consuming—probably even more time-consuming than quantitative selection. And they could prove more opaque, possibly more open to manipulation. But at best, they would improve efficiency in the selection process.

Looking at NPM's economism as a whole, the obvious thing to do is to stabilize the academic job market. To increase output through outsourcing or otherwise precarious working conditions is a fallacy. Good science requires what might be called calm waters: conditions that inspire creativity and intelligence, not inhibit them. The argument can be extended: beyond good working conditions in general, it can also make sense to create a certain free space that is not subject to evaluation. By this, of course, I do not mean pausing ethical guidelines, but rather a leeway of resources. This free space can also be abused, of course, and that has been a past experience. On the other hand, under the influence of NPM, it is clear that an excess of evaluation is also an obstacle to the scientific process. A certain degree of trust—specifically, a certain amount of basic funding—seems to make functional sense and is at the same time part of (ethically) good working conditions. In the end, this is about the academic institution's trust in itself: anyone who has made it to the PhD with above-average success should in principle be able to conduct worthwhile research projects. Ultimately, all this is a reflection of the fact that scientific breakthroughs cannot be planned. They happen unexpectedly, and that is precisely the functional advantage of free science.

## Résumé

With the perspective developed, we can address problems of NPM from a socio-philosophical setting.

Based on a remark by Habermas, we had initially followed an intuition, which can be described with the scheme inside/outside. Academy organizes itself—then something comes from the outside and reorganizes it according to external standards. As we have seen, however, this intuition is not that convincing. First, it is not unproblematic from a methodological point of view. Second, it provides only limited thrust for the critique of NPM.

My proposal starts with orienting the idea of a proprietary logic rather to the social ontology of forms of life, i.e., to conceive it in normative-functionalist terms. That is, to simultaneously relate the proprietary logic of academic institutions back to a broader understanding of what it means to function well (also ethically). With this twist, the focus shifts from provenance to the applicability of NPM to academic organizational problems. Can NPM help academy function well?

So, we had to deal with this sub-question materially. In doing so, we found that NPM connects to problems, but often solves them unsatisfactorily, or creates new problems in an attempt to solve them.

From these challenges, we can indirectly, and of course tentatively, deduce what future organizational forms will have to deal with. On the one hand, qualitative criteria could find a stronger consideration in evaluation. The challenge here is to implement these criteria in a way that they are transparent and resistant to abuse. On the other hand, it could prove useful to suspend a certain area from evaluation—in the sense of an institutionally secured, creative leeway. Here, one of the challenges would be to combine this area with evaluation-based research funding in a fruitful way.

## Data Availability Statement

The original contributions presented in the study are included in the article/supplementary material, further inquiries can be directed to the corresponding author/s.

## Author Contributions

The author confirms being the sole contributor of this work and has approved it for publication.

## Conflict of Interest

The author declares that the research was conducted in the absence of any commercial or financial relationships that could be construed as a potential conflict of interest.

## Publisher's Note

All claims expressed in this article are solely those of the authors and do not necessarily represent those of their affiliated organizations, or those of the publisher, the editors and the reviewers. Any product that may be evaluated in this article, or claim that may be made by its manufacturer, is not guaranteed or endorsed by the publisher.

## Footnotes

^1^Taken from Kraan et al.'s teaser (flap text).

^2^Cf. (as his magnum opus in social theory) Habermas, (1984/1987) [1981]).

^3^I am adopting here, as already touched upon in the abstract, a rather essayistic form, if you will. The approach could be labeled that way in contrast to, for example, common methodological assumptions of Analytical Philosophy in the narrower sense. At this point, only so much: The form is intended to follow what might metaphorically be called a ‘movement in thought'. It aims at connections rather than at the definition of concepts and their conditional structures (cf. Adorno, 1984).

^4^I am summarizing here with Kraan a line that has already been resumed by Hood: “The doctrinces … encompassed by NPM have been variously described … there is still a god deal of overlap … shift … from policy making to management … stress on output … competitive bias … variable pay … more emphasis on contract provision” (Hood, 1995, p. 95).

^5^This is also related to the fact that the distinction between a management cadre (those who implement NPM) and scientists is not always clear-cut. Of course, it is the case that management and science policy bear more responsibility for the developments described than do ‘ordinary' researchers. It is important to point this out because the distinction is rarely made in what follows. There are two reasons for that. First, the socio-philosophical resources drawn upon focus mainly on social ensembles. On the other hand, and with this I return to the first sentence, it seems to me that there are also gray areas (not all scientists consider NPM problematic, or they might have mixed opinions, and act accordingly).

^6^Cf. (mainly) Habermas, (1988) [1976].

^7^Cf. in particular chapter 10: “Self-Reflection as Science: Freud's Psychoanalytic Critique of Meaning”.

^8^Cf. Habermas, (1987) [1972], Appendix (“Knowledge and Human Interest. A General Perspective”).

^9^Cf. (Habermas, (1984/1987) [1981]), also (commenting) Strecker (2017).

^10^Cf. Kruse (2020).

^11^To give at least the probably most urgent further hints: earlier Habermas “discusses tendencies whereby democratic self-determination comes to be replaced by technocratic administration … In this context, he offers considerations of the ‘practical consequences of scientific-technological progress,' which he seeks to distance from the optimistic, liberal view of technology as a means of unburdening humanity (and a controlled means, at that); equally, he seeks to qualify overly grim assessments by cultural critics and the overenthusiastic fanfares of technocrats” (Celikates and Jaeggi, 2017, p. 261). In the context of *Communicative Rationality*, this balanced stance continues: “the development of steering mechanisms (such as power and money) […] make[s] action coordination possible without having to rely on mutual understanding. These alternative mechanisms of action coordination help preserve social order by reducing the risk of dissent” (Lafont, 2017, p. 298) — yet only as long as “alternative steering mechanisms” do not “penetrate—and shape—the lifeworld”. Back then, “Habermas offers a two-tiered response to this problem. The first involves supporting communicative action institutionally by embedding it in the law, and the second the radical proceduralization of legitimate rule” (Deitelhoff, 2017, p. 528).

^12^Cf. Habermas, (1988) [1976], p. 71.

^13^Cf. Strecker (2017, p. 370): “Freed from the demand for understanding (*Verständigung*), the realms of material social reproduction uncouple themselves from the lifeworld, which in turn yields the private and public spheres; henceforth, the lifeworld encompasses only the symbolic reproduction of society. Accordingly, Habermas describes social development as a two-step process of differentiation”.

^14^Cf. Kruse (2022).

^15^Cf. Chandler et al. (2002, p. 1061): “NPM has successfully colonized the academy”.

^16^Cf. Vrouwe (2010).

^17^Since we started with Habermas, it should not go unmentioned that Habermas also argues in some places (e.g., in *Knowledge and Human Interest's* appendix) against such a “l'art pour l'art.” Husserl presents a particularly interesting position in this context. Husserl misunderstands himself in Habermas' eyes. His phenomenology is supposed to allow “pure theory” through strict contemplation. But this “pure theory”, precisely because it is “pure”, is supposed to promote certain empirical interests. Thus, seen in this way, it is rather an indirect argument – structurally similar to the central one in Kant's Aesthetics.

^18^Cf. Jaeggi (2018, p. 100ff).

^19^Cf. Jaeggi (2018, p. 145ff).

^20^Cf. Jaeggi (2018, p. 246ff).

^21^Cf. Jaeggi (2018, p. 173ff).

^22^Of course, with these in the end cursory remarks, I do not want to hide the fact that Jaeggi's theory may offer the mentioned conclusions, but has not (yet) systematically elaborated every aspect. In the CFL there is, so to speak, (no more, but also no less than) the layout of a ‘full' theory of society.

^23^To anticipate an obvious question: in Jaeggi's case, then, is it not ultimately also true that the immanent standards of well-functioning are overwritten by external ones (those of NPM), and that critique ultimately finds its footing in there? This question leads to the subtle but at the same time momentous shifts in Critical Theory's recent history. What is correct, in my view, is that Habermas' and Jaeggi's approaches repeatedly come very close to each other. The most relevant difference for us can be illuminated, for example, by their relationship to Hegel. Habermas considers Hegel's objections against Kant plausible in some points, but does not see his theory neuralgically affected by them (cf. Habermas, (1989) [1986]). Jaeggi, on the other hand, moves in many respects on the tracks of a theory of ethical life [Sittlichkeit]. This means for us: From “boundary conditions” (such as conditions of redeemability of morality, if norms can be realized or even be understood) cannot be abstracted easily. In a sense, they are always a (more or less important) part of a whole. In short, Jaeggi is less concerned with socio-ontological division in the strong meaning of the term. The CFL is, in a way, transversal to it. What appears from another perspective as interaction of social subsystems is with her more of a question of scale and overlap.

^24^Taken from Kraan et al.'s teaser (flap text, see above).
